# Extraction, Preparation and Characterization of Nanocrystalline Cellulose from Lignocellulosic Simpor Leaf Residue

**DOI:** 10.3390/molecules30071622

**Published:** 2025-04-05

**Authors:** Ukashat Mamudu, Asset Kabyshev, Kenzhebatyr Bekmyrza, Kairat A. Kuterbekov, Aliya Baratova, Lukman Ahmed Omeiza, Ren Chong Lim

**Affiliations:** 1 Material Analysis and Metal Testing Department, National Metallurgical Development Centre, Jos PMB 2116, Nigeria; ukashatmamudu87@yahoo.com; 2 Faculty of Physics and Technical Sciences, L. N. Gumilyov Eurasian National University, Astana 010008, Kazakhstan; 3 Faculty of Integrated Technologies, Universiti Brunei Darussalam, Jalan Tungku Link, Gadong BE1410, Brunei; 4 Centre for Advanced Material and Energy Sciences, Universiti Brunei Darussalam, Jalan Tungku Link, Gadong BE1410, Brunei

**Keywords:** nanocrystalline cellulose, lignocellulosic, simpor leaf residue, sulfuric acid hydrolysis, α-cellulose, phytocompounds

## Abstract

In this study, α-cellulose was extracted from lignocellulosic simpor leaf residue as a sustainable alternative to conventional cellulose sources. The extraction process involved the removal of hemicellulose, lignin, and other phytocompounds using alkali (NaOH) treatment and bleaching with hydrogen peroxide (H_2_O_2_). The nanocrystalline cellulose (NCC) was isolated from α-cellulose using sulfuric acid hydrolysis treatment followed by ultrasonication. The extracted α-cellulose and isolated NCC were characterized using Fourier transform infrared spectroscopy (FT-IR), X-ray diffraction (XRD), thermogravimetric analysis (TGA), and dynamic light scattering (DLS). The obtained results confirmed that the extracted NCC exhibited characteristic cellulose functional groups and a crystallinity index of 64.7%, indicating the effective removal of amorphous regions through sulfuric acid hydrolysis. The thermal stability of the extracted cellulose increased to 332 °C due to the elimination of extractives. DLS analysis showed that the extracted NCC exhibited high colloidal stability in polar solvents, characterized by a zeta potential of −70.8 mV and an average particle size of 251.7 nm. This study highlights an environmentally friendly approach for converting low-value biomass waste into high-value cellulose materials with potential applications in sustainable packaging, biomedical applications and composite reinforcement.

## 1. Introduction

There are vast sources of sustainable materials on Earth that are underutilized. Among them, lignocellulosic biomass holds immense potential for the extraction of biopolymers/nanomaterials [1,2]. Plant leaves, as part of this biomass, are deployed for the production of green polymers and bio-based materials in the pharmaceutical industry [3]. Also, extracts from these leaves (as green corrosion inhibitors) have attracted tremendous research attention in formulating corrosion inhibition for metallic infrastructures in various aggressive environments. For example, the simpor *(Dillenia suffruticosa)* plant belongs to the family of *Dilleniaceae*, an evergreen plant in Southeast Asia that grows quickly, spreads, and survives in most tropical environments [4]. Leaves from this plant are composed of phytocompounds with proven efficacy in traditional wound healing [5] and the corrosion inhibition of metals in harsh environments [6]. After the extraction of these phytocompounds, the lignocellulosic biomass is discarded as waste. However, this biomass can be valorized by the separation of different biopolymers such as lignin and cellulose [7]. Thus, this research work is focused on the recycling of the lignocellulosic biomass in cellulose production.

Cellulose, the most abundant biopolymer on Earth, is produced at an estimated 1.5 × 10^12^ tons annually [8]. It is a primary component of the cell wall that offers support and structural rigidity to the plant. Being a linear homopolymer, cellulose is composed of crystalline (highly ordered) and amorphous (disordered) regions. As the dominant constituent of plant fibers, cellulose consists of multiple β-D-glucose units linked by β-1,4-glycosidic bonds, forming highly linear chains. While it exhibits a crystalline structure that is insoluble in water, the remaining components are amorphous and easily permeable [8,9]. Its high mechanical strength, water absorptivity, tunable physicochemical properties and biodegradability renewed its interest in academia and industry [10].

Many studies have explored the extraction of cellulose from plant-based resources such as stalks, barks, shafts, and stems [11,12,13]. In the work of Md Salim et al., cellulose was obtained from *Leucaena leucocephala* bark using ethanol–toluene solvent extraction. The phytocompounds were first removed, followed by cellulose extraction through alkali treatment and bleaching [3]. Similarly, Bellesia et al. investigated cellulose extraction from giant cane (GC), *Posidonia oceanica* seagrass (PO) plants, coffee silverskin (CS), and brewer’s spent grain (BSG) residues. The authors followed a three-step extraction process: phytocompound and ash removal using an ethanol solution; hemicellulose and lignin removal via alkali treatment; and final bleaching with NaClO_2_ solution. The characterization of the extracted cellulose using Fourier transform infrared (FTIR) spectroscopy, nuclear magnetic resonance (NMR) spectroscopy, X-ray diffraction (XRD), and scanning electron microscopy (SEM) confirmed its high purity [10]. In another study, Bolio-López et al. extracted cellulose type I from *Calathea lutea* fibers using alkali treatment, bleaching, and acid hydrolysis. FTIR analysis confirmed the successful removal of lignin and hemicellulose, while XRD analysis revealed that the extracted cellulose fibers were predominantly amorphous with small crystallite sizes [14]. Additionally, Al-Dabash and Al-Kahali extracted nanocellulose from banana pseudostem fiber through degumming, delignification, and sulfuric acid hydrolysis. The nanocellulose was extensively characterized using FTIR spectroscopy, transmission electron microscopy (TEM), energy dispersive X-ray (EDX) analysis, field emission scanning electron microscopy (FE-SEM), and Brunauer–Emmett–Teller (BET) analysis. The study further explored the application of the extracted nanocellulose in the removal of Pb and Cd ions from aqueous solutions [15].

The world’s total amount of above-ground biomass (woody) in forests is 4.2 × 10^11^ tons [16]. Plant leaves contained average of 10% to 12% of cellulose [17]. Despite the cellulosic content of plant leaves, their potential as a source for cellulose extraction remains largely unexplored in cellulose production. This study aimed to isolate α-cellulose from lignocellulosic simpor leaf residue, a Soxhlet extraction by-product of green corrosion inhibitors. Alkali and bleaching treatments were deployed to obtain α-cellulose that had undergone sulfuric acid hydrolysis to produce nanocrystalline cellulose (NCC) [1]. The precursor (lignocellulosic simpor leaf residue and α-cellulose) and NCC were further characterized using Fourier transform infrared (FTIR) spectroscopy, X-ray diffraction (XRD), thermogravimetric (TGA) analysis and dynamic light scattering (DLS). This study offers the potential of plant leaves as a sustainable source of cellulose, as the quest for renewable materials production from agricultural waste intensifies. In addition to nanocellulose applications, the development of advanced functional materials from biomass and mineral precursors has attracted growing attention in energy-related technologies. For instance, novel perovskite-type materials such asNd_1-x_Sr_x_Mn_0.5_Cr_0.5_O_3-δ_, synthesized through solid-state reactions, demonstrate high electrochemical stability, electrical conductivity, and compatibility with common electrolytes for solid oxide fuel cells (SOFCs), making them promising for green hydrogen energy applications. A detailed method for their preparation is described in a recent utility model [18].

## 2. Results and Discussion

### 2.1. Functional Groups Characterization

**Figure 1:**
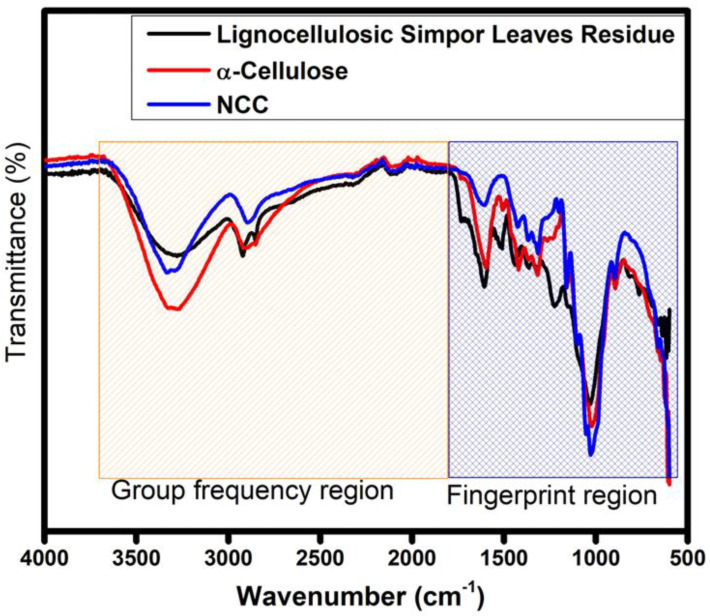
FTIR spectra of lignocellulosic simpor leaf residue, α-cellulose and NCC, showing group frequency and fingerprint regions.

The FTIR spectra of lignocellulosic simpor leaf residue, α-cellulose and NCC samples are shown in Figure 1. In the spectra, overlapped peaks demonstrate the presence of cellulose. Peaks at the group frequency region (1800–4000 cm^−1^) were broad, and those at the fingerprint region (600–1800 cm^−1^) exhibited higher intensity and sharper peaks in the spectra of α-cellulose and NCC [19]. However, in the spectrum of simpor leaf residue, peaks at 1606 cm^−1^ and 1218 cm^−1^ showed higher intensities, with the latter peak eliminated upon alkali, beaching and acid hydrolysis treatment, as indicated in the NCC spectrum. A similar observation was previously reported study during cellulose extraction from *Leucaena leucocephala* bark [3].

In the spectra, the observed changes in functional groups indicated changes in chemical structure due to the alkali, bleaching and sulfuric acid hydrolysis treatments of the lignocellulosic simpor leave residue. The broad peaks between 2985 cm^−1^ and 3656 cm^−1^ are assigned to O-H groups, and 2887 cm^−1^ signifies the existence of C-H stretching bonds. The spectrum of simpor residue showed an additional peak of asymmetry of the methylene group, which was also eliminated after the treatments, indicating the removal of phytochemical constituents from the residue. The fingerprint region of all the spectra exhibited overlapping peaks that affirmed the existence of a cellulose structure in the simpor leaf residue.

#### 2.1.1. Functional Groups of Lignocellulosic Simpor Leaves Residue

**Figure 2:**
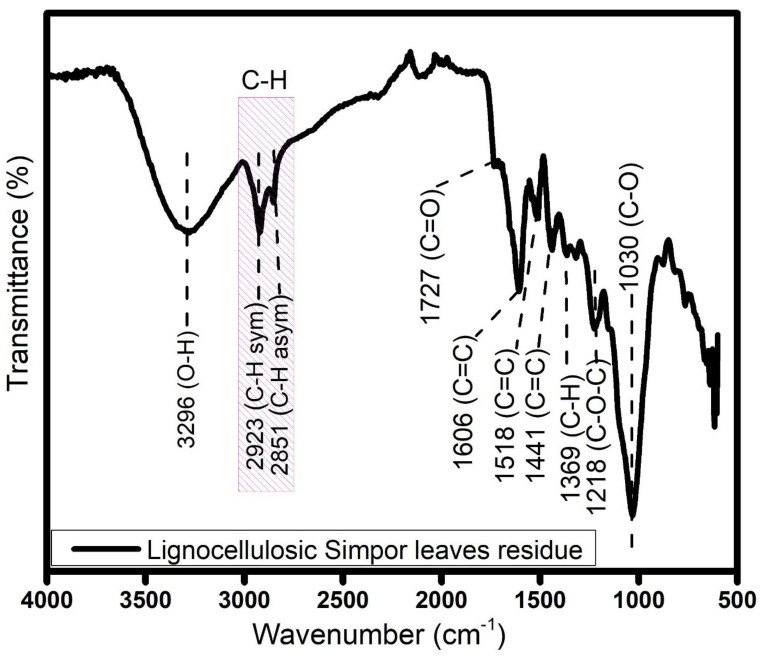
FTIR spectra of lignocellulosic simpor leaf residue.

In Figure 2 the FTIR spectrum shows functional groups of lignocellulosic simpor leaf residue.

The broad peak at 3296 cm^−1^ is assigned to an O-H stretch vibration in polyphenol in the simpor leaves. The peaks at 2923 and 2851 cm^−1^ are attributed to the symmetry and asymmetry of C-H stretching vibrations. Next to these peak positions is the carbonyl group (C=O) peak at 1727 cm^−1^

This peak indicates acetyl and ester groups in hemicellulose or carboxylic acid groups in the ferulic and p-coumaric components of lignin. This peak was similarly observed for untreated rice husk and corn stalk, respectively, in the extraction of cellulose [20,21]. The peak at 1606 cm^−1^ indicates the C=C group of the oligosaccharide linkage absorption to sapogenins, which is related to the saponin constituent of the simpor leaves [22]. The peaks at 1518 cm^−1^ and 1441 cm^−1^ were reported as C=C stretching in the aromatic ring, which are attributed to extractives in the simpor leave residue [3]. Cellulose in this residue was further demonstrated with the characteristic peak at 1369 cm^−1^, which is typically attributed to the C-H deformation vibrations within the cellulose molecule. The presence of peaks at 1218 and 1030 cm^−1^ indicated C-O-C and aliphatic C-O stretching vibrations [23]. The peak at 1031 cm^−1^ is assigned to the C–O–C group of the oligosaccharide linkage absorption to sapogenins [4].

#### 2.1.2. Functional Groups of α-Cellulose

**Figure 3:**
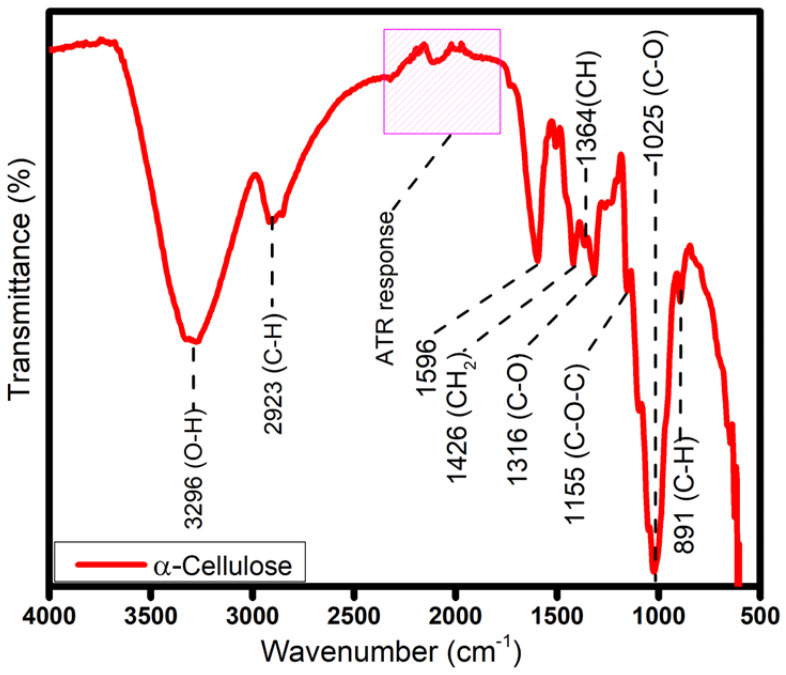
FTIR spectra of α-cellulose.

The FTIR spectrum of α-cellulose after alkali-bleaching treatments of lignocellulosic simpor leaf residue is shown in Figure 3. In the spectrum, the removal of hemicellulose and lignin suggested the disappearance of peaks between 1727 cm^−1^ and 1441 cm^−1^. The characteristic peaks of cellulose were observed between 1426 cm^−1^ and 800 cm^−1^. Typical to pure cellulose are the peaks at 1426, 1365, 1315, 1155, 1025 and 891 cm^−1^, which are evident in the spectrum.

The peak at 1426 cm^−1^ corresponds to the C-O-H in-plane bending vibration at C6. The peaks at 891 and 1155 cm^−1^ are related to the C-O-C stretching at the β-1,4-glycosidic linkages of the anhydroglucose unit (AGU) in the molecular structure of cellulose [24]. The appearance of these peaks in the spectrum suggested that alkali treatment and bleaching can suitably remove hemicelluloses, lignin, and other extractives to produce cellulose from simpor leaf residue.

#### 2.1.3. Functional Groups of NCC

**Figure 4:**
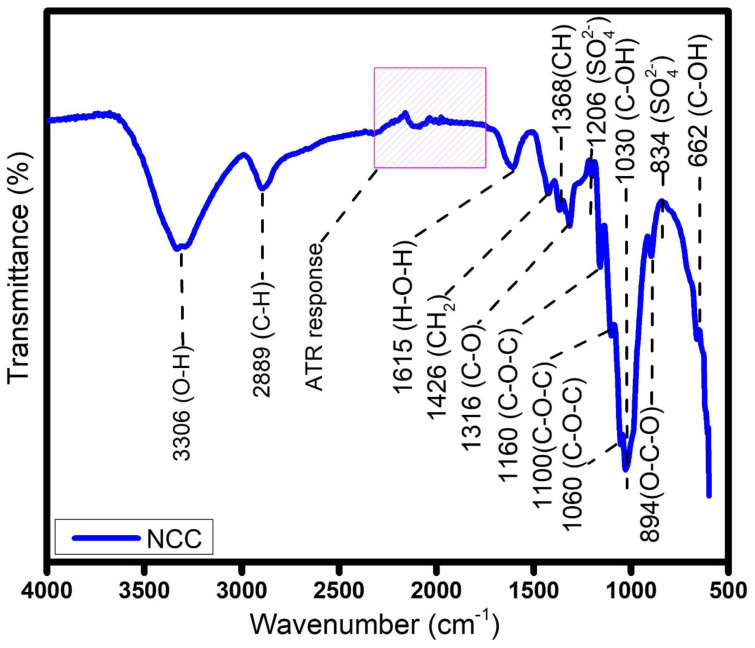
FTIR spectra of NCC.

It is demonstrated in Figure 4 that the sulfuric acid hydrolysis of α-cellulose does not change the structure of cellulose, aside from the formation of sulfate ester groups that enhance the stability of the isolated NCC in polar solutions. In addition to the characteristic peaks of cellulose shown in the spectrum of α-cellulose, the NCC spectrum (in Figure 4) demonstrates peaks at 1636 cm^−1^, 1160 cm^−1^, 1100 cm^−1^, 1060 cm^−1^, 894 cm^−1^ and 662 cm^−1^, which are typical for pure cellulose. From these, the peak at 1636 cm^−1^ is attributed to the O-H bond of the adsorbed water molecules on the cellulose [25]. As previously mentioned, the peak at 1426 cm^−1^ indicated the C-OH in-plane bending vibration at C6. As illustrated in Figure 1, the changes in the environment at C6 during sulfuric acid hydrolysis caused the reduction of the intensity of the peak at 1426 cm^−1^, observed in the NCC spectrum, in comparison to the α-cellulose [26]. The hydroxyl groups at C6 are easily hydrolyzed due to the steric hindrance [2]. During the sulfuric acid hydrolysis of the α-cellulose, these hydroxyl groups are esterified to the sulfate ester groups, and the changes in the environment lead to the reduction in the intensity of the peak corresponding to C-OH.

The peaks at 1060, 1100 and 1160 cm^−1^ are related to the C-O-C pyranose ring stretching vibration. These peaks are prominent in the NCC spectrum, compared to the α-cellulose. The peak at 894 cm^−1^ corresponds to the cellulosic β-glycosidic linkages attributed to O-C-O stretching [24]. The appearance of these peaks in the spectrum suggests that alkali treatment and bleaching suitably extracted hemicelluloses, lignin, and other extractives from the simpor leaf residue to produce cellulose. The sulfuric acid hydrolysis of this cellulose does not change its structure, aside from the formation of sulfate ester groups that enhanced the stability of the isolated NCC in polar solutions. The peaks at 834 cm^−1^ and 1206 cm^−1^ are assigned to these sulfate groups.

### 2.2. Crystal Structure Characterization

Cellulose’s structure is composed of crystalline and amorphous structures, unlike hemicellulose and lignin, which are wholly/completely amorphous. The crystalline structure in cellulose is attributed to the hydrogen bonding interactions and Van der Waals forces between adjacent molecules [21]. The crystalline nature of NCC after alkali, bleaching and sulfuric acid hydrolysis treatments of the simpor leaf residue was evaluated using X-ray diffraction (XRD) analysis. Based on the XRD spectra, it is evident that alkali, bleaching and sulfuric acid hydrolysis treatments of the simpor leaf residue can destroy the amorphous region, thus increasing the crystallinity of the extracted cellulose (α-cellulose and NCC).

**Figure 5:**
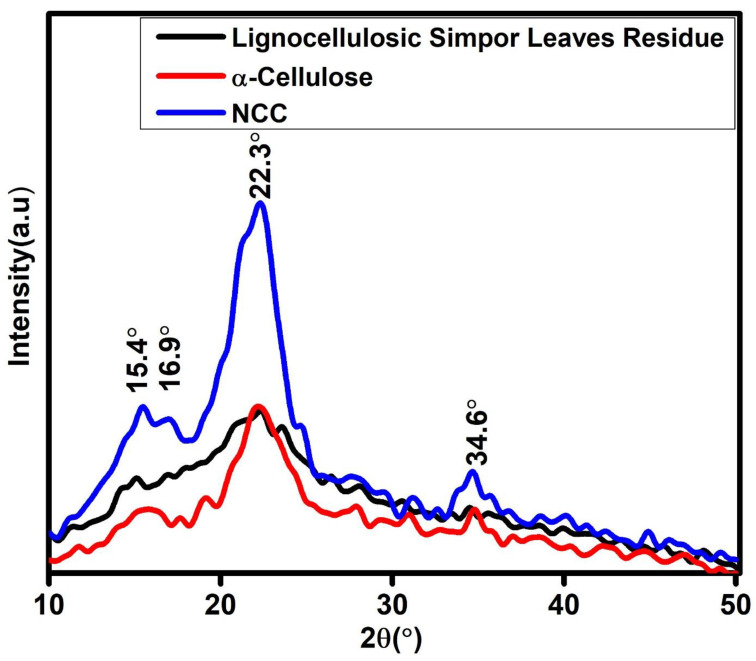
XRD spectra of lignocellulosic simpor leaf residue, α-cellulose and NCC.

The diffraction peaks of lignocellulosic simpor leaf residue, α-cellulose and NCC obtained from the XRD analysis are shown in Figure 5. These peaks have crystal planes of [1 1 0], [1 1 0], and [2 0 0] that correspond to the 2θ = 15.4°, 16.9°, and 22.3° of the monoclinic cellulose Iβ lattice. The crystal planes and 2θ positions suggest that the α-cellulose and NCC were cellulose type I [20]. These peaks refer to more defined NCC due to the removal of the amorphous region during sulfuric acid hydrolysis.

The changes in the crystallinity index and crystallite sizes of the most intense peak of the lignocellulosic simpor leaf residue, α-cellulose and NCC are summarized in Table 1. In the case of NCC, the crystallinity index significantly increased to 64.7% after alkali, bleaching and sulfuric acid hydrolysis treatments. The increased crystallinity index can be attributed to the partial removal of extractives (hemicellulose, lignin and remaining phytocompounds) and the amorphous fraction of cellulose by chemical treatments [27]. This result corroborated with a reported study in the literature in which the extraction of cellulose from rice husk was performed [21]. During sulfuric acid hydrolysis, hydronium ions attack amorphous regions of the cellulose, leading to the cleavage of oxygen in the glycosidic link of each glucan unit and releasing individual cellulose crystals. The growth and realignment of these crystals enhance the cellulose crystallinity, as demonstrated in the narrowing of the XRD peak of NCC in Figure 5. The crystallinity index of NCC obtained in this study is within the range of previously reported work in the literature, as presented in Table 2.

The broad peaks at 2θ = 22.3°demonstrated the highest intensity in the XRD spectra of lignocellulosic simpor leaf residue, α-cellulose and NCC. The broadest peak was observed for the lignocellulosic simpor leaf residue which indicated a smaller crystallite size as illustrated in Table 2. The crystallite size increased from 0.67 to 1.67 after the alkali and bleaching treatments of this residue. After the sulfuric acid hydrolysis of the α-cellulose, this crystallite size slightly increased to 1.82, demonstrated in the case of NCC. This result corroborated the crystallite size reported for nanocellulose prepared from microcrystalline cellulose using alkali and urea treatments [28].

### 2.3. Thermal Stability Analysis

**Figure 6:**
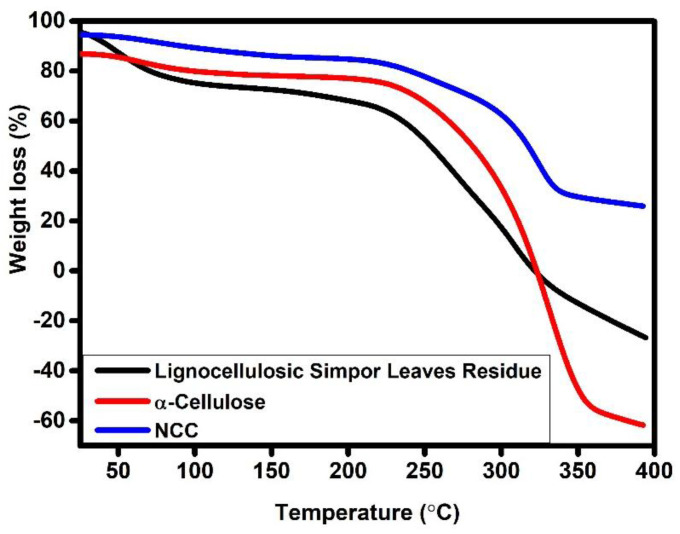
TGA.

The thermal stability of cellulosic material is influenced by its inherent characteristics and the molecular interactions between its macromolecules. When the applied thermal energy exceeds the bond dissociation energy of specific chemical bonds, macromolecular chains undergo cleavage or bond dissociation [27]. TGA was performed to assess the thermal stability of lignocellulosic simpor leaf residue, α-cellulose and NCC. In Figure 6, the TGA and derivative thermogravimetry (DTG) curves correspond to the weight loss and derivative weight of the cellulose samples upon continuous heating from 25 °C to 400 °C. In the TGA curves, the lignocellulosic simpor leaf residue showed three peaks of thermal degradation around 50 °C, 267 °C and 315 °C. Similarly, α-cellulose showed three peaks around 65 °C, 250 °C and 332 °C, while NCC demonstrated peaks around 90, 250, and 325 °C. The first peak observed between 25 °C and 100 °C is ascribed to moisture evaporation and the loss of some volatile components from the samples during heating. The lignocellulosic simpor leaf residue, α-cellulose and NCC demonstrated average mass loss values of 20%, 7%, and 5%, respectively, due to absorbed moisture, within this temperature range. The next regions in the TGA curve are from 100 °C to 190 °C, 290 °C and 288 °C for simpor leaf residue, α-cellulose and NCC, respectively. This region is considered the thermal stability zone; beyond these temperatures, thermal degradation begins for the simpor leaf residue, α-cellulose and NCC samples.

In the thermogram of simpor leaf residue, the second peak at 267 °C corresponds to the degradation of hemicelluloses. These hemicelluloses are thermally less stable due to their structural characteristics. Within this temperature range, lignin and cellulose also thermally degrade at lower rates. The third peak of simpor leaf residue is approximately 315 °C and is attributed to cellulose degradation. It is noteworthy that cellulose is more resistant to thermal degradation among the extractives in the simpor leaf residue and is responsible for about half of the dry biomass composition, which explains the higher peak intensity in the DTG graph at about 340 °C [29].

**Figure 7:**
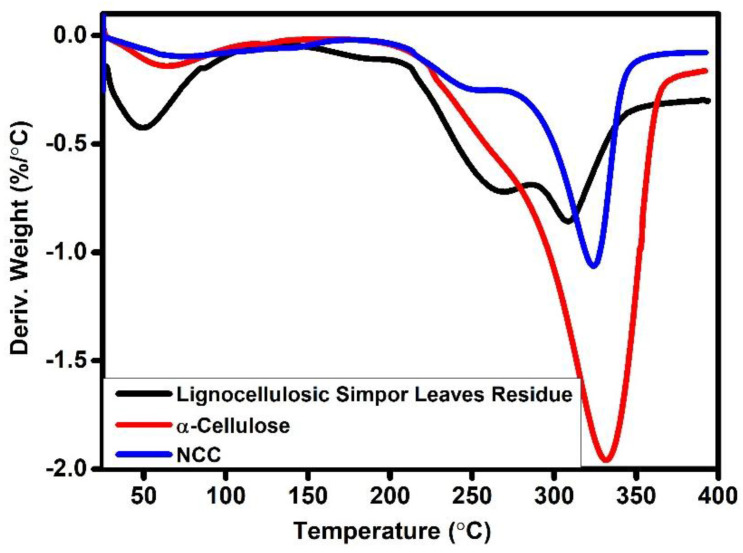
DTG.

In Table 2, the thermal degradation (DTG) of the samples occurred in the range of 315–332 °C, which was mainly due to the thermal degradation of cellulosic materials. After alkali and bleaching treatments of the simpor residue, the thermal degradation temperature shifted from 315 °C to 332 °C in the case of α-cellulose (Figure 7. This increasing degradation temperature can be attributed to the removal of extractives (hemicellulose, lignin and remaining phytocompounds) and the amorphous fraction by alkali and bleaching treatment of the residue, thus increasing the crystallinity content of the α-cellulose. After the sulfuric acid hydrolysis treatment of the α-cellulose, the resulting NCC formed demonstrated a slight reduction in the degradation temperature to 325 °C. This reduction in the degradation temperature of NCC, despite its higher crystallinity content, can be attributed/ascribed to the thermal degradation of the sulfate groups formed on the NCC surface during acid hydrolysis. Moreover, the lower degradation temperature could also be correlated to the reduced length of NCC with respect to α-cellulose. The TGA results exhibited that the thermal stability of the extracted α-cellulose and isolated NCC increased compared to the simpor leaf residue.

### 2.4. DLS Results

The DLS measurement was unsuitable for evaluating the zeta potential and average particle size of the simpor leaf residue and α-cellulose samples as their particles flocculate after suspension in solvents. The flocculation of these samples could be ascribed to a higher force of attraction between the particles compared to the repulsive forces [30]. As presented in Table 2, the NCC sample has a zeta potential and average particle size of −70.8 mV and 251.7 nm. This zeta potential value indicated stronger repulsive forces between adjacent charged particles, thus promoting the dispersion of NCC in the solvent. The negative zeta potential value indicates negatively charged anions (sulfate groups) on the NCC surface, which enhances the electrostatic layer on the NCC surface [31]. The sulfate groups were formed during sulfuric acid hydrolysis, where hydroxyl groups on the α-cellulose undergo esterification, forming sulfate ester groups in the extracted NCC, as shown in Figure 5. Therefore, sulfuric acid hydrolysis of the α-cellulose could effectively produce NCC that is stable in polar solvents. This is unlike the zeta potential value of −37.0 mV obtained during phosphoric acid hydrolysis of cellulose [32]. The higher zeta potential value of −70.8 mV for this NCC indicates an increased surface charge due to the grafting of half-ester sulfate groups onto the highly reactive hydroxyl groups (C_6_–OH). This high zeta potential absolute value of more than 30 mV indicates that NCC has a sufficient repulsive force that could cause its stability in the polar solvents.

## 3. Materials and Methods

### 3.1. Materials

Analytical-grade sulfuric acid (95–97%) was procured from Merck, Darmstadt, Germany. Hydrogen peroxide (H_2_O_2_) and sodium hydroxide (NaOH) pellets were purchased from Fisher Scientific, Waltham, MA, USA, and Chameleon Reagent, Hiratsuka, Japan. Scharlau, Shanghai, China, supplied absolute ethanol. Simpor leaves were sourced from Universiti Brunei Darussalam Botanical Garden, Brunei Darussalam. All chemicals were used as received without modifications, and deionized water was prepared in our laboratory (Table 3).

### 3.2. Extraction of Cellulose from Simpor Leaves Residue

**Figure 8:**
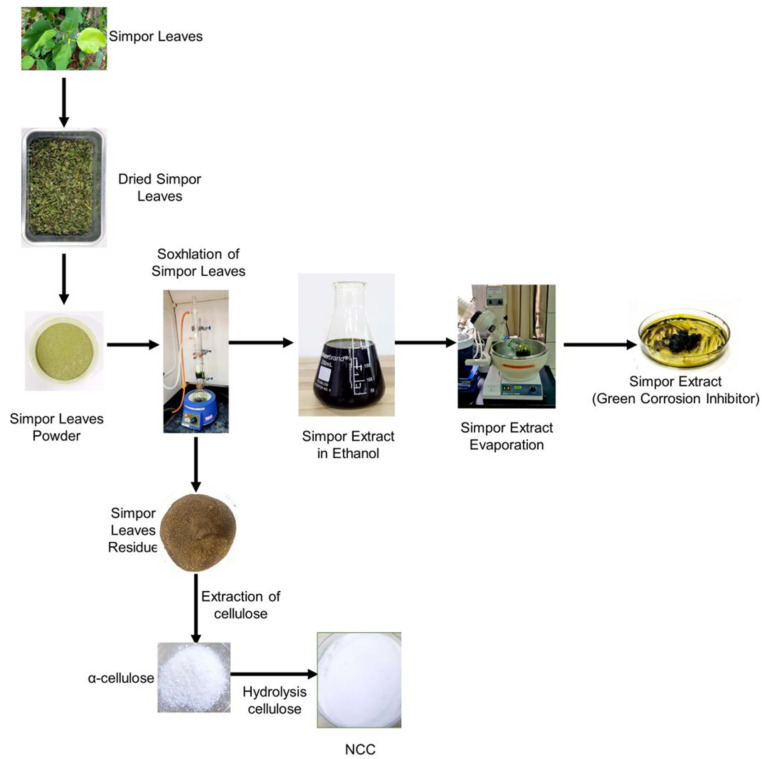
Schematic of cellulose extraction from lignocellulosic simpor leaf residue.

Fresh simpor leaves were washed in a flowing tap and rinsed with double-deionized water to remove dirt. The leaves were dried in the oven at 40 °C for three days. After that, the dried leaves were ground into powder and admitted into a cellulose extraction thimble. The simpor extract was obtained from the powder using Soxhlet extraction equipment. This extraction process was performed at 79 °C for 4 h, with absolute ethanol as the solvent. The extract solution was concentrated into a dark-green viscous substance using a rotary evaporator and deployed as a green corrosion inhibitor [4]. The lignocellulosic simpor leaf residue was collected and processed for cellulose extraction as shown in the schematic in Figure 8.

### 3.3. Extraction of Cellulose from Lignocellulosic Simpor Leaves Residue

In a beaker containing 300 mL of 1 m sodium hydroxide (NaOH) solution, 40 g of lignocellulosic simpor leaf residue was added and stirred at 80 °C for 30 min in a magnetic stirrer. Notably, alkali (NaOH) treatment was performed to increase the stiffness while removing impurities and producing α-cellulose [3]. The mixture was centrifuged at 4000 rpm for 10 min to wash off the alkali solution. Furthermore, the washing process was repeated four times with distilled water at 4000 rpm for 5 min. The supernatant was discarded, while the lignocellulosic simpor leaf residue was collected and bleached in 200 mL of 30 *v*/*v*% hydrogen peroxide (H_2_O_2_) at 80 °C for 30 min, with slow stirring at 900 rpm in a magnetic stirrer.

The resulting solution was cooled down and filtered with Whiteman filter paper, and the lignocellulosic residue was collected and allowed to remain in the hydrogen peroxide solution for 24 h to ensure the complete removal of chromophores and lignin compounds in the simpor leaf residue [33]. The suspension was centrifuged at 4000 rpm for 15 min to remove the peroxide. The supernatant was decanted, and the extracted cellulose fibers were dissolved in ethanol and washed at 4000 rpm for 20 min until a pH of 4.5 was obtained. The extracted α-cellulose fibers were dried at 80 °C to a constant weight before sulfuric acid hydrolysis was performed.

### 3.4. Isolation of Nanocrystalline Cellulose (NCC)

Nanocrystalline cellulose (NCC) was isolated by sulfuric acid hydrolysis after drying the extracted α-cellulose fibers at 80 °C to a constant weight. This hydrolysis was carried out using the method previously reported [2]. The method involves adding extracted α-cellulose into a pre-heated 40% sulfuric acid (H_2_SO_4_) solution at an acid-to-cellulose ratio (ACR) of 10:1. Sulfuric acid was used for this hydrolysis as the process produced NCCs of uniform particle dimensions. Moreover, this acid could functionalize the cellulose (NCC) surfaces with about 0.25 mmol/g of sulfate groups, which enhances their stability in polar solvents [2]. The suspension was stirred in a magnetic stirrer at 60 °C for 45 min. The hydrolysis was stopped by adding cold distilled water at 4°C and centrifuging at 4000 rpm for 10 min. The hydrolyzed α-cellulose was collected and dissolved in H_2_O_2_ and sonicated for 20 min at 37 kHz, 100 Watts, in an ultrasonic cleaner (Elmasonic P60H), Elma, Singen am Hohentwiel, Germany. The suspension was centrifuged at 4000 rpm for 10 min to remove H_2_O_2_. The residue was collected, dissolved in absolute ethanol and centrifuged repeatedly until a pH of 5.5 was achieved. The NCC was dried at 80 °C for FTIR and XRD analysis.

### 3.5. Characterizations

#### 3.5.1. Fourier Transform Infrared (FTIR) Spectroscopy Analysis

The chemical structure of the lignocellulosic simpor leaf residue, α-cellulose and NCC was established using Shimadzu attenuated total reflectance–Fourier transform infrared (ATR-FTIR) spectrometer, Kyoto, Japan. This analysis was conducted within a spectra range of 4000–600 cm^−1^ at a resolution of 4.0 cm^−1^, with 32 total scans. The lignocellulosic simpor leaf residue, dried α-cellulose and NCC were placed on the diamond crystal of the equipment one at a time, and the knob was used to press the samples against the crystal. At the same time, the spectrum was recorded in transmittance mode as a function of the wavenumber. 

#### 3.5.2. X-Ray Diffraction (XRD) Analysis

The crystal structures of lignocellulosic simpor leaf residue, α-cellulose and NCC were analyzed using the Shimadzu XRD-7000 X-ray diffractometer, Kyoto, Japan. The XRD patterns were collected using monochromatic Cu Kα-radiation (λ = 0.15406 nm) at operating conditions of 40 kV, 30 mA, and a scan rate of 5°/min, with a 2θ range from 10° to 50° for cellulose samples. From the XRD spectra, the crystallinity index (C.I) and crystallite size were obtained to describe the cellulose structure.

The C.I describes the amount of crystalline cellulose present in the cellulose structure due to the removal of hemicellulose, lignin, and amorphous fraction during alkali and acid hydrolysis treatment of lignocellulosic simpor leaf residue and α-cellulose, respectively. We have previously reported Segal’s equation in evaluating the crystallinity index of cellulose [32]. The method is quick and straightforward, comparing peak heights at crystalline and amorphous regions, corresponding to 2θ positions on the spectra, as shown in Equation (1).

(1) $$CI=\frac{I_{200}-I_{amorp}}{I_{200}}\times 100\%$$

where I_amorp_ is the intensity of diffraction peaks of the amorphous region at a 2θ angle and I_200_ is the intensity of diffraction peak at the crystalline region ([2 0 0] lattice plane) at a 2θ angle of about 22.7°.

However, the crystalline fraction of cellulose is proportional to the area of the crystal peaks rather than the heights. Thus, Segal’s equation does not accurately describe the level of crystallinity of this cellulose [34]. Hence, we adopted the area-under-peaks method to calculate the crystallinity index of the cellulose samples. This method compares the area under crystalline peaks to the total area under both crystalline and amorphous peaks in the spectrum. By the area method, we calculated the crystallinity index using Equation (2).

(2) $$C.I=\frac{Area\;under\;crystaline\;peaks}{Area\;under\;both\;crystalline\;and\;amorphous\;peaks}\times 100\%$$

Crystallite size is another essential parameter that describes the effect of alkali acid hydrolysis treatment on the crystalline structure of the cellulose on the [1 1 0], [1 1 0], and [2 0 0] lattice planes. This is obtained from Scherrer’s equation.

(3) $$\mathrm{Crystallite}\;\mathrm{size}\;(D_{\mathrm{hkl}})=\frac{k\lambda }{\beta cosӨ}$$

where Scherrer’s constant (k) is 0.94, X-ray radiation wavelength (λ) is 0.15406 nm, and the full width at half maximum (FWHM) of the diffraction peak (β) in radians is half of the diffraction angle. The FWHM was determined from OriginPro 9.0 software using nonlinear curve fit and the Lorentzian function [2].

#### 3.5.3. Thermogravimetric (TGA) Analysis

Thermogravimetric analysis (TGA) was performed to analyze the thermal stability of lignocellulosic simpor leaf residue, α-cellulose and NCC using a Thermogravimetric analyzer, TGA Q500, (TA Instruments, New Castle, DE, USA). The samples (10 mg) were placed in a platinum pan in a nitrogen environment purged at 50 mL/min and heated within the temperature range of 25–400 °C at a ramping temperature of 10 °C/min. The onset degradation temperature (T_o_), residual weight (W_residue_) and maximum temperature of degradation (T_max_) were obtained from the TGA analysis.

#### 3.5.4. Dynamic Light Scattering Experiment

A dynamic light scattering (DLS) experiment was carried out to measure the average particle size and zeta potential of NCC. The suspension was prepared by dispersing 10 mg of NCC in 100 mL of distilled water and sonicated for 60 s. The average particle size and zeta potential of NCC in the suspension were measured in triplicate at room temperature using the Malvern 3000 Zetasizer Nano ZS instruments, Worcestershire, United Kingdom.

## 4. Conclusions

This study successfully extracted nanocrystalline cellulose (NCC) from lignocellulosic simpor (Dillenia) leaf residue through a series of alkali, bleaching, and acid hydrolysis treatments. The removal of hemicellulose and lignin was confirmed by the disappearance of characteristic peaks in the FTIR spectrum. The extracted NCC demonstrated good crystallinity, stability in polar solvents, and enhanced thermal stability after the efficient removal of amorphous regions by sulfuric acid hydrolysis. The enhanced properties of the extracted NCC could promote their deployments in bio-based coatings, biodegradable packaging, or drug delivery systems. Further work would involve studying their morphology (using SEM and TEM) and quantifying the sulfate content introduced due to hydrolysis treatment. The findings from this study highlight the potential of simpor leaf residue as a sustainable alternative to conventional cellulose sources, contributing to the valorization of agricultural waste into high-value nanomaterials with promising industrial applications.

## Figures and Tables

**Table 1:** Crystallinity index and crystallite size, thermogravimetric and DLS results of lignocellulosic simpor leaf residue, α-cellulose and NCC.

Sample	XRD Results		Thermogravimetric Results		DLS Results	
	Crystallinity Index (C.I) (%)	Crystalline Size of D_200_ (nm)	TGA (T_O_ (°C))	DTG (T_max_ (°C))	Zeta Potential (mV)	Z-Average Particle Size (nm)
Simpor leaf residue	51.3	0.67	230	315	-	-
α-cellulose	52.4	1.67	290	332	-	-
NCC	64.7	1.82	288	325	−70.8	251.7

**Table 2:** Crystallinity index of NCC extracted from other plant resources.

Biomass	Extraction Protocol	Acid Concentration	Hydrolysis Time (Minutes)	Hydrolysis Temperature (°C)	Crystallinity Index (%)	Reference
Dried stalk of ***Corchorus olitorius***	Acid hydrolysis	1 M sulfuric acid	35	90	88.32	[12]
Sugarcane bagasse	Acid hydrolysis	64% *w*/*w* sulfuric acid	45	55	77.	[13].
Pineapple crown leaf fiber	Acid hydrolysis	1 M sulfuric acid	60	45	63.34	[1]
Ananas comosus leaf wastes	Acid hydrolysis	40% *w*/*w* sulfuric acid	25	45	75.89	[11]
Rice husk	Acid hydrolysis	10 molL^−1^ sulfuric acid	40	50	59.0	[23]
Simpor leaf residue	Acid hydrolysis	40% *w*/*w* sulfuric acid	45	60	64.7	This study

**Table 3:** A summary of chemicals, equipment, and roles in cellulose and NCC extraction process.

List of Chemicals and Equipment	Roles
Hydrogen peroxide (H_2_O_2_)	Bleaching
Sodium hydroxide (NaOH)	Increase stiffness of cellulose while removing impurities such as lignin and hemicellulose
Absolute ethanol	Washing of extractives from cellulose
Analytical grade of sulfuric acid	Hydrolysis of cellulose
Soxhlet apparatus	phytocompounds removal from the residue
Oven	Drying of the extracted cellulose and NCC

## Data Availability

The original contributions presented in this study are included in the article. Further inquiries can be directed to the corresponding authors.
